# Residual effect of vermicompost and preceding groundnut on soil fertility and associated *Striga* density under sorghum cropping in Eastern Ethiopia

**DOI:** 10.1371/journal.pone.0318057

**Published:** 2025-03-12

**Authors:** Addisu F. Ebbisa, Nigussie Dechassa, Zelalem Bekeko, Feyera Liben

**Affiliations:** 1 School of Plant Sciences, Haramaya University, Dire Dawa, Ethiopia; 2 Ethiopia Institute of Agricultural Research, Addis Ababa, Ethiopia; 3 Alliance of Biodiversity International and CIAT, ILRI, Addis Ababa, Ethiopia; University of Lucknow, INDIA

## Abstract

Depletion of soil organic matter was found to be the primary biophysical factor causing declining per capita food production in sub-Saharan Africa. The magnitude of this problem was exacerbated by moisture-stress and imbalanced fertilizer application that caused *Striga* weed infestation. To address such confounded issues, two-year field experiments were conducted to evaluate the effect of residual vermicompost and preceding groundnut on soil fertility, sorghum yield, and *Striga* density. The first-year treatments contained two sowing methods (single and intercropped sorghum), two seedbed types (open-furrow and tied-ridge), and four vermicompost rates (0, 1.5, 3.0, and 4.5 t/ha) combined factorially in a randomized block design. In the second-year experiment, only monocropped sorghum with seedbed types was sown exactly on the same plot as the previous year’s treatment combinations without fertilizer. The results disclosed that residual vermicompost at 4.5 t/ha in intercropped sorghum/groundnut significantly reduced soil pH (0.76%), bulk density (8.61%), electrical conductivity (38.78%), and *Striga* density (85.71%). In contrast, compared to unamended soil, the aforementioned treatment combined with tied-ridging increased soil moisture, organic matter, and sorghum yield by 16.67, 2.34, and 58%, respectively. Moreover, this treatment combination markedly increased post-harvest soil organic carbon (7.69%), total N (0.247%), available P (38.46%), exchangeable-Fe (27%), and exchangeable-Zn (40%) in the second year over control. Treatments previously amended with 4.5 t/ha of vermicompost under the sorghum-groundnut intercrop system resulted in the highest total N (0.242%) and available P (9.822 mg/Kg). Thus, the vermicompost and groundnut successfully improve soil fertility and sorghum yield for two cropping seasons.

## Introduction

Poor soil fertility is one of the greatest biophysical constraints adversely affecting the food security of millions in sub-Saharan Africa (SSA) [1,2]. These problems were highly exacerbated by unbalanced fertilizer application [3,4], severe depletion of soil organic matter (SOM) [5,6], and overuse of crop residues for home consumption instead of as fertilizer [2,7,8]. According to Beshir & Abdulkerim [9], the situation was predominantly noticeable in Ethiopian dryland regions, which exacerbated the spread and tenacity of the *Striga hermonthica* (Del.) Benth weed infestation in SSA [10,11]. This disastrous weed regulates the flow of water and nutrients from the host to itself using an organ known as haustoria [10,12,13]. Numerous studies suggested that the combination of improved soil fertility with *Striga*-resistant varieties was one of the sustainable ways to overcome the menace of *Striga* weed [14–17]. Similarly, to stifle this parasitic weed, there was a high possibility of replacing escalating chemical fertilizer prices with low-cost and high-quality organic matter [1,18,19]. High-quality organic matter could be used for soil bioremediation via modifying soil rhizosphere, promoting microbial diversity, and enriching soil enzymes [20,21]. This enhances host-plant resistance and/or tolerance in post-attachment stages [17,22]. SOM is also used as the reservoir of essential plant nutrients [23,24] that had a high residual (carryover) effect for the following crops [25] and may last for many years [26].

In this scenario, the application of vermicompost was suggested as the most promising and eco-friendly natural fertilizer [21,27,28] that could mitigate soil degradation and replenish SOM [23,29]. The most described features that make vermicompost an effective fertilizer is its high homogeneity, porosity, water-holding capacity, readily available plant nutrients, and low C:N ratio [30–32]. Providing an optimal nutrient level, could correct “*Liebig’s Law of the Minimum*” and mitigate the high cost of chemical fertilizer [20,21], resulting in high agronomic, economic, and environmental benefits [33]. Following the Law of the Minimum, vermicompost is beneficial not only for crop nutrition but also for reducing plant constraints on other resources like water, disease, plant growth hormone, etc. [32]. This vermicompost would be easily prepared at low cost [34] from locally affordable raw materials such as *Catha edulis Forsk* [35], *Parthenium hysterophorus* L. [36,37]*, Lantana camara* L. [38,39], *Prosopis Juliflora*, etc. [40]. Furthermore, the use of vermicompost has multifold benefits, such as tackling the competing issue (frequent removal) of farmyard manure (FYM) and crop residue [7,8]. It also creates new job opportunities for rural households, particularly for women [38–42]. *P*. *hysterophorus* [40] and *L. camara* [38] are the most invasive and problematic weeds in terrestrial ecosystems that have greatly disturbed human welfare.

Currently, the distribution of these notorious weeds is spreading rapidly and extensively [43,44] thereby results in 97-100% yield reductions of field crops in eastern and southern Africa [45]. Management of these weeds through mechanical, biological, and chemical means was too difficult and ineffective [39,43,46]. Direct incorporation of their green matter (residues) also poses detrimental effects on crops and soil health due to its toxicity [47]. Additionally, these low-quality organic materials immobilized soil nutrients, prolonging the soil infertility cycle [2]. Thus, management of these notorious weeds through utilization, particularly by vermicomposting, is the only sustainable way to destroy their allelopathic (*phenolics and sesquiterpene lactones*) and toxic properties [37,40,47,48].

Nevertheless, the decomposition and nutrient-releasing rate of applied vermicompost in dryland agriculture were predominantly influenced by soil moisture stress [20,49] that negatively affected microbial growth and nutrient diffusion [50,51]. In this regard, employing tied-ridge tillage [20,50–53] with a cereal-legume intercropping system would effectively conserve soil moisture and assist rate of decomposition and nutrient release [9,54,55]. According to Cong *et al*. [56], intercropping hastens SOM decomposition, probably associated with a lower soil C:N ratio, higher litter input, a change in the rhizosphere system, and better N retention. Besides this, incorporating legumes in cereal crops delivers additional income, nutritional benefits, and N to the soil system [1], while reducing *Striga* weed pressure [3]. Intercropping systems strongly influenced soil microbial diversity, structure, and function [57,58], besides improving plant growth traits [59], which was conducive to improving soil nutrient-supplying capacity and microecosystem stability [60,61].

Furthermore, intercropping exhibited high ground-cover due to more shading compared to sole cropping, thus lowering soil evaporation and increasing the SMC [62]. Studies have also shown that legume crops have substantially residual benefits for cereals [63]. The authors further found that the total amount of nitrogen carried over from diverse legumes to successive crops in soybean, peanut, and chickpea ranged between 54 and 68 Kg per ha. On the other hand, a tied-ridge substantial improves soil water by decreasing surface water flow velocity and bulk density [64]. These practices aid in reducing the spread of *Striga* weed to other fields [14,65,66]. However, the integrated use of vermicompost and tied-ridging under a sorghum-intercropping system was poorly understood despite being of valuable importance in protecting plants from *Striga* weed invasion and water stress. As per Ekeleme *et al*. [67] and Yang *et al*. [68], the majority of the evidence that is currently available about the impact of vermicompost on soil physico-chemical parameters was obtained in a controlled environment. Apart from this, there was meager information on the effects of the preceding groundnut and leftover vermicompost on *Striga* density. Liu *et al*. [69] also noted the effect of preceding legumes on subsequent crop yield, and soil has not been well explored.

Determining the target nutrient needed for a subsequent crop begins with understanding the soil’s fertility status and the residual effects of applied fertilizer. The effective application of this technology therefore needed a deeper comprehension of such synergistic impacts on soil physico-chemical characteristics. Moreover, Erkossa *et al*. [4] state that more comprehensive efforts were required to raise knowledge in order to distract farmers from the usage of synthetic fertilizer, which was previously supported by Ethiopian government subsidies. Hence, we hypothesize that the residual effect of vermicompost combined with a tied-ridge under the sorghum/groundnut intercropping system would resolve the most yield-limiting factors such as soil infertility, moisture stress, SOM depletion [5,6], and severe *Striga* infestation [10,11]. Thus, we intended to evaluate the residual effects of vermicompost and preceding groundnut intercropped with sorghum under various seedbed types on soil physio-chemical properties, *Striga* density, and sorghum yield. Subsequently, we aimed to control the most destructive invasive weeds via the vermicomposting process.

## Materials and methods

### Site description

**Fig 1:**
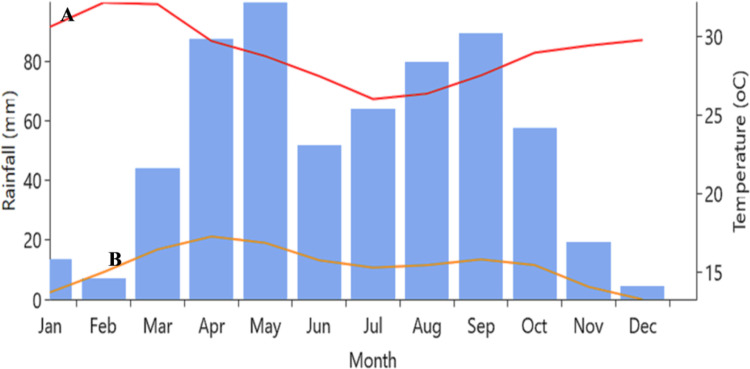
Long-term (1991–2022) monthly rainfall (bar), maximum (A), and minimum (B) temperature at Kile site.

The experiment was conducted in a farmers’ field at Kile-Besidimo Plain in the 2021 and 2022 years. Kile-Besidimo is one of the smallest farmers administrative associations in the Sofi district of Harari Region in eastern Ethiopia. This research was approved by Haramaya University (HrU) Post Graduate Director and Office of Research Affairs, with special responsibility given to the “PES Research Thematic Area Leaders,” for directing field site and project-related activities. The geographical location of the site is 42^0^ 27’ N latitude and 9^0^ 12’ E longitudes and an altitude of 1400 meters above sea level. The district has a bimodal rainfall distribution pattern with heavy rains in April and May, while the long and erratic rainy month stretches from June to October (Fig 1). The average annual rainfall amount is 249.2 mm, and the maximum and minimum temperatures are 21.9°C and 10.6°C, respectively (Fig 1). The site was known for its monoculture cropping history, particularly sorghum.

### Vermicompost preparation

The fresh biomass of L. *camara*, P. *hysterophorus*, and animal dung as well as dried wheat straw was collected from HrU and its vicinity areas. After the plant residues were chopped into small pieces (2–3 cm), each substrate was weighed in an equal amount (3 Kg) and mixed all together, then fresh animal dung was added in a 1:2 ratio of dry plant residue on a weight basis. The mixture was moistened and turned manually every day for three weeks as pre-composting (partial fermentation) in a previously constructed vermiculture house at the HrU Raree Research Site. This vermiculture station comprises rooms for worm raring, substrate preparation, and vermicomposting, as well as drying and storage with sufficient shade and ventilation. This process made feeds more biodegradable and palatable to worms [21]. Young *Eisenia fetida* were added to mixed substrate filled in a worm bin according to the recommendation in proportion by Shirani *et al*. [30], in which 25 g of earthworms were added per 1 Kg of cow manure. Beside their fecundity and suitability, *Eisenia fetida*, so-called eco-biological engineer [70], consumes organic matter equal to their body weight every day [71]. The mixture was maintained moist by spraying water every five days to regulate the worm bins optimum range of temperature (15-25°C), moisture (65-75%), and pH (6.5-7.2). The matured vermicompost was harvested, air dried, and added to determine its chemical properties, ready for use as fertilizer.

### Treatments and experimental procedures

Field experiments were conducted for two consecutive years, 2021 and 2022, using two crop cultivars: ‘*Melkam* (WSV387)’ for sorghum [*Sorghum Bicolor* (L.) Moench] as a base crop and ‘*Babile-1* (ICGV-98412)’ for groundnut (*Arachis hypogaea* L.) as a component crop. The first-year trial consisted of sole and intercropped sorghum with groundnut combined with/without (i) two seedbed types (open-furrow and tied-ridge) and (ii) four vermicompost rates (0, 1.5, 3.0, and 4.5 t/ha), which were factorially arranged using a randomized complete block design with three replications (Table 1). These vermicompost rates were calculated based on previously recommended fertilizer in the sole crop method for sorghum [72] and groundnut [73] in the study area. We also take into consideration the negative effect of excess vermicompost on salinity and N_2_-fixation suggested by Oyege & Bhaskar [21].

**Table 1:** Treatment combinations in the first-year and second-year cropping systems for sorghum.

	First (2021) year Treatments				Second (2022) year Treatments		
No	Combinations	**No**	Combinations	**No**	Combinations	**No**	Combinations
1	SSFV_0_	**13**	SB_1_FV_0_	**1**	**SSF**_**0**_	**13**	**SS**_**1**_ **F**_**0**_
2	SSFV_1_	**14**	SB_1_FV_1_	**2**	SSF_1_	**14**	SS_1_F_1_
3	SSFV_2_	**15**	SB_1_FV_2_	**3**	SSF_2_	**15**	SS_1_F_2_
4	SSFV_3_	**16**	SB_1_FV_3_	**4**	SSF_3_	**16**	SS_1_F_3_
5	SSTdV_**0**_	**17**	SB_1_T_d_V_0_	**5**	SSTd_0_	17	SS_1_T_d0_
6	SSTdV_1_	**18**	SB_1_T_d_V_1_	**6**	SSTd_1_	18	SS_1_T_d1_
7	SSTdV_2_	**19**	SB_1_T_d_V_2_	**7**	SSTd_2_	**19**	SS_1_T_d2_
8	SSTdV_3_	**20**	SB_1_T_d_V_3_	**8**	SSTd_3_	**20**	SS_1_T_d3_

SS=sole sorghum; SB = intercropped sorghum with B_1_ = *Babile-1* or B_2_ = *Babile-2*; V =  vermicompost; Td = tied-ridge; F = furrow-planting (conventional planting), SS_1_ = sole cropped sorghum in previous *Babile-1*; F _(0-4)_ =  sorghum in furrow planting under previous applied rates of vermicompost; Td _(0-4)_ =  sorghum in tied-ridging under previous applied rates of vermicompost.

Tied-ridge is a type of *in-situ* soil moisture conservation tillage in which a ridge of 20 cm high with 3-5 cm thickness was created with a ridge maker fitted on a tractor and was tied at 1.5-m intervals manually [74]. The crossties are kept lower than the ridges, so they act as spillways in the event of heavy rainfall to reduce soil erosion. These micro-basin tillage types increase surface retention capacity and nutrient availability via reducing runoff and nutrient losses from soil, thereby improving soil moisture content while reducing *Striga* dissemination to neighboring fields. Open-furrow is a shallow trench or row formed by conventional farmer practice (oxen-drawn traditional *maresha*), which is a bit deeper than a flatbed and shallower than a tied-ridge.

In the first main cropping season, the land was plowed and harrowed by a tractor to bring the soil to a fine tilth, and then sorghum and peanut were sown on 28 June 2021. In the second year, the land was carefully prepared using a spade without destroying and mixing the previous plot size as well as plot shape (Table 1). Under this year, only sorghum and seedbed types without fertilizer was sown on June 2, 2022, using the same plot and randomization of its first-year treatment combination. In all intercropping treatments, the row spacing between sorghum and groundnut was maintained at 37.5 cm with a net plot area of 3 m x 4 m (12 m^2^), with the space between each plot and the block remaining 1.0 m and 1.5 m, respectively. For the tied-ridge planting pattern, sorghum seeds were dribbled in the row of ridges, while groundnut seeds were planted on the ridges between the sorghum rows. Vermicompost was incorporated into the soil only in the first-year trial according to a predetermined rate to each plot a week before sowing. To keep the field weed-free except for *Striga*, manual weeding and hoeing operations were performed during both years of experimentation. All other necessary agronomic management practices were carried out uniformly as per standard recommendation for sorghum and groundnut cultivation.

### Sample collection

#### Physico-chemical properties of soil and vermicompost.

For assessment of the fertility status of soils in the study area, a composite soil sample from a depth of 0–20 cm was taken at the beginning of the trial (2020) from the whole field as well as immediately after harvest of first-year (2021) and second-year (2022) crops from each plot. For better results, one composite sample was prepared for laboratory analysis from 4 sampling points per plot, avoiding the border effect. The physico-chemical properties of soil, as well as vermicompost that was free from earthworms and cocoons were analyzed at the HrU soil laboratory for specific parameters. Soil pH by potentiometric water extract (pH-H_2_O), electrical conductivity (EC mS/cm), total N (%) by the Kjeldahl method available P by the Olsen method cation exchange capacity (CEC), exchangeable bases (Ca, Mg, K, and Na) by ammonium acetate, micronutrients (Zn, Fe, and Mn) by the *Ethylenediamine Tetra-acetic Acid* (EDTA) extraction method, and total organic carbon by the Walkley and Black method. Soil bulk density and moisture content were measured from undisturbed soil by the core method and gravimetric method, respectively. The weight of the wet soil was measured immediately after collecting the sample, while the dry weight of each sample was measured after oven drying at 105°C for 24 hours for both parameters. Moisture content was determined at the vegetative and early reproductive stages (during the 50% sorghum flowering stage) just after two days of rainfall. The following formula was used for calculating the soil moisture content (SMC).


$$SMC=\frac{Ww-Wd}{Wd}*100$$

Where: SMC =  soil moisture content dry base (%), Ww =  weight of the wet soil (gm), Wd =  weight of the dry soil (gm).

#### *Striga* variable.

The number of emerging *Striga* plants in each plot was counted at intervals of two weeks [77, 91, 105, and 119 days after sowing (DAS)] from the four center rows of each net plot (3.4 m^2^) until all *Striga* matured to determine the maximum above-ground *Striga* number. *Striga* density was calculated following Nkoa *et al*. [75] and Travlos *et al*. [76] steps. It is one of the simplest and most common means of computing weed abundance [75].


$$\text{Striga density}=\frac{Total\text{Striga count per plot }}{\text{Harvested plot area }\left(m^{2}\right)}$$

Where: High *Striga* density indicates severe infestation.

#### Sorghum parameters.

Stover yield and grain yield were measured after harvest from the net plot area (by discarding external rows). Following the adjustment of the moisture content to 12.5%, the grain yield of sorghum was measured and translated to tons per hectare (t/ha).

### Data analysis

Selected soil physico-chemical, *Striga* density, and sorghum yield data were subjected to analysis of variance (ANOVA) using R-Software (R-4.3.1 version). In all the comparisons, the level of significance was set at α =  0.05. The hypotheses were tested at a 95% confidence interval, and the means were compared using the least significant difference (LSD) test.

## Results

### Quality of vermicompost and soil before planting

The result of laboratory analysis of vermicompost obtained from a mixture of all substrate materials had a neutral pH (7.1), high organic carbon (23.67%), an acceptable C:N ratio (12.24%), and medium EC (5.32 mS/cm) (Table 2). This compost also contains a higher percentage of essential plant nutrients than the initial soil analysis result (Table 2). At the beginning of the experiment, total N, available P, as well as exchangeable K, Mg, Ca, and organic matter in the top 30 cm soil layer were 0.01%, 3.48 mg Kg^-1^, 0.45 cmol (+)/Kg, 3.75 cmol (+)/Kg, 9.92 cmol (+)/Kg, and 1.08%, respectively. Correspondingly, initial soil results at the experimental site had a high bulk density (4.5 g cm^-3^), a medium CEC (18.17 cmol (+)/Kg), and low Zn and Fe contents.

**Table 2:** Preliminary physico-chemical properties of vermicompost and pre-treated soil.

Properties	Parameter values		Properties		Parameter values
	VC	Soil		VC	Soil
pH (H_2_O)	7.25	7.1	Mn (mg/Kg)	19.467	1.3
EC (mS/cm)	5.32	3.33	Exch. Ca (cmol (++)/Kg)	32.77	9.92
Total N (%)	1.93	0.01	Exch. Mg (cmol (++)/Kg)	25.848	3.75
OM (%)	40.72	1.08	Exch. K (cmol (+)/Kg)	20.14	0.45
Av. P (mg/Kg)	20.14	3.48	CEC (cmol (+)/Kg)	43.5	18.17
Zn (mg/Kg)	44.42	0.19	Sand (%)	–	66.78
Fe (mg/Kg)	54.33	5.83	Clay (%)	–	15.06
BD (g/cm^3^)	–	1.45 (compact)	Silt (%)	–	18.16

VC = vermicompost; N =  total nitrogen; OM =  organic matter; Av.P =  available phosphorus; Zn =  zinc; Fe =  iron; Mn =  manganese; Exch =  exchangeable cations (K =  potassium, Mg =  magnesium, and Ca =  calcium); CEC =  cation exchange capacity; EC =  electrical conductivity; BD =  soil bulk density.

### Effect of residual vermicompost and groundnut on selected soil physico-chemical characteristics at Kile

#### Soil organic carbon (SOC) and bulk density (BD).

**Fig 2:**
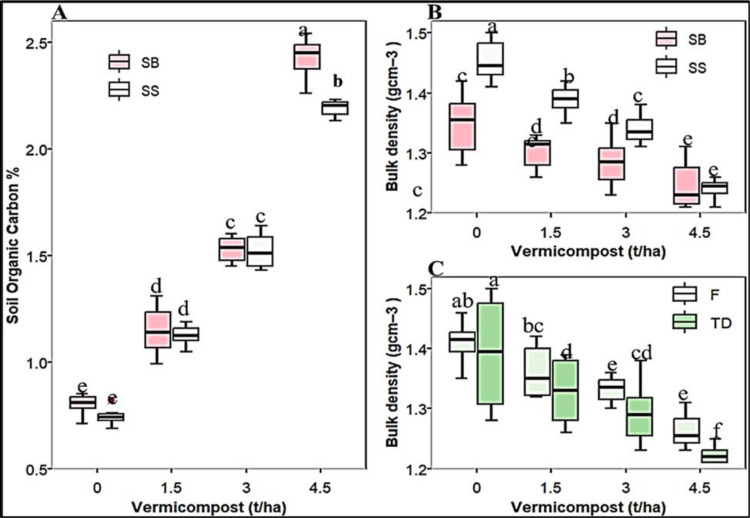
Joint interaction effect of vermicompost and planting methods on soil organic carbon (A) and bulk density **(B)**, as well as two-way interaction effect of vermicompost and seedbed types on soil BD **(C)**; “SS,” “SB,” “F,” and “TD” stand for “sole sorghum,” “intercropped sorghum with groundnut,” “flat bed,” and “tied ridge,” respectively. Boxes sharing the same letter under each figure were statistically similar.

The mean separation of the second-year post-harvest analyzed soil in Table 3 indicates that the single effects of the three factors, as well as their two-way interaction with vermicompost, significantly (*P* ≤ 0.05) affect soil BD (S2 Table in S1 File). However, only the main effects of the planting method and vermicompost, along with their two-way interaction, show significant differences for SOC (Table 3). The highest SOM matter (4.472%) was recorded at 4.5 t/ha vermicompost applied to previous sorghum/groundnut intercropping, while the lowest (1.02%) was obtained in the control plot (Fig 2A). A similar trend was seen in SOC (Fig 2A). Additionally, as the rate of vermicompost increased from nil to 4.5 t/ha under the intercropping system, the soil SOC increased by 51.28% and SOM by 88.2% (Fig 2A). Application of high vermicompost dosage reduces soil compaction by 8.61% than the control plot. The minimum soil BD (1.22 g/cm³) was recorded at 4.5 t/ha of vermicompost combined with the tied-ridge planting pattern (Fig 2C), while the maximum (1.45 g/cm³) was observed in monocropped sorghum without any treatments (Fig 2B). Increasing the rate of vermicompost decreased soil bulk density by 7.81% and 7.22% under both sowing methods and seedbed types compared to the corresponding control (Figs 2B and C).

**Table 3:** Second-year post-harvest mean squares of ANOVA of soil physico-chemical properties at Kile in 2022 cropping seasons.

Source of Variation	df	Organic carbon	Bulk density	pH	CEC	EC	Nitrogen(N)		Phosphorus (P)		Potassium (K)
**SM**	1	0.08**	0.43**	0.05**	28.08*	85.23*	0. 0019*		1.62*		0.14**
**SB**	1	0.005^ns^	0.13**	0.025*	8.68^ns^	264*	0. 0004^ns^		1.91*		0.01*
**VC**	3	1.19**	0.05**	0.27**	422**	2874*	.054**		77**		1.15**
SM*SB	1	0.001^ns^	0.001^ns^	0.01^ns^	8.39^ns^	11.19^ns^	0.00^ns^		0.01^ns^		0.002^ns^
SM*VC	3	0.03*	0.01**	0.02*	43.6**	47.11*	0. 001**		0.38*		0.04**
SB*VC	3	0.001^ns^	0.007*	0.02**	11.33*	61.24*	0. 0007*		0.62*		0.003^ns^
SM*SB * **VC**	3	0.001^ns^	0.001^ns^	0.003^ns^	2.20^ns^	11.69^ns^	0. 0002^ns^		0.11^ns^		0.001^ns^
**Errors**	47	34.8	0.001	0.001	3.08	5.8	0. 0001		0.11		0.002
**CV (%)**		**5.56**	**5.86**	**5.88**	**7.93**	**5.52**	**7.77**		**5.87**		**7.01**
**Source of Variation**	**Df**	**Calcium (Ca)**	**Iron (Fe)**	**Zinc (Zn)**	**Manganese (Mn)**	**SMC1**	**SMC2**	**Striga Density**		**Stover-yield (t/ha)**	**Grain yield (t/ha)**
**SM**	1	1.21^ns^	61.6*	0.12**	0.62**	83.9**	31.23	60.55**		2.76*	1.14**
**SB**	1	0.02^ns^	7.23^ns^	0.01^ns^	0.07^ns^	538**	121**	61.22**		19.594**	4.35**
**VC**	3	194**	21**	3.65**	0.21**	244**	148**	97.38**		70.94**	23.21**
SM*SB	1	1.43^ns^	6.03^ns^	0.13**	0.06^ns^	97.36**	6.68*	4.71**		0.332^ns^	0.3^ns^
SM*VC	3	4.04*	8.39*	0.024*	0.08*	6.16*	8.08**	2.86**		0.784^ns^	0.05^ns^
SB*VC	3	0.05^ns^	0.07^ns^	0.03*	0.001^ns^	35.75**	8.42**	2.78**		1.083*	0.52*
SM*SB * **VC**	3	1.62^ns^	1.8^ns^	0.017*	0.02^ns^	10.42*	9.63**	1.36**		1.787*	0.29*
**Errors**	47	0.87	2	0.004	0.02	1.80	1.23	0.13		0.362	0.08
**CV (%)**		**6.59**	**6.87**	**6.01**	**6.87**	**6.73**	**7.03**	**7.05**		**9.668**	**8.89**

df = degree of freedom; ns =  not significant;

* and

**=  significant at 5% and 1% probability levels, respectively. CEC =  cation exchangeable capacity; EC =  electric conductivity; SMC =  soil moisture content at vegetative (1) and reproductive stage (2); sowing methods (SM); seedbed type (SB); VC =  vermicompost; CV =  coefficient of variation.

#### Soil pH, cation exchange capacity (CEC), and electrical conductivity (EC).

**Fig 3:**
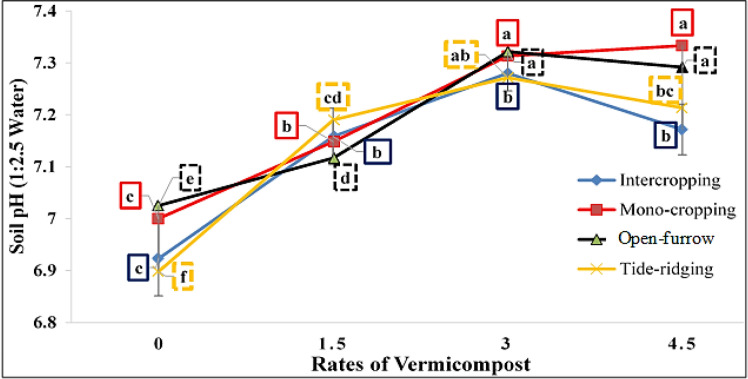
Two-way interaction effect of vermicompost and planting methods on soil pH. Line sharing the same letter was statistically similar.

The main effects and two-way interactions of vermicompost and cropping systems significantly (*P* ≤ 0.05) affected soil pH, CEC, and EC (S3 Table in S1 File), while the three-way interaction effect and the two-way interaction effect between planting and seedbed types were insignificant (Table 3). Preliminary soil analysis shows that the soil pH is approximately 1.79% lower than the residual vermicompost at a rate of 3 t/ha in the second cropping season (Fig 3). Increasing the rates of vermicompost from nil to 3 t/ha under intercropping raised soil pH by 2.54%, but at 4.5 t/ha, it decreased by 0.76% (Fig 3). Likewise, soil pH increased with higher rates of vermicompost up to 3 t/ha by 2.02% and 2.68% under flat-bed and tied-ridge planting patterns, respectively, with a decreasing trend at the highest fertilizer dose (Fig 3). Consequently, the highest CEC (33.362 cmol (+)/Kg) was recorded at 4.5 t/ha of vermicompost under sorghum-groundnut intercropping, followed by the open-furrow planting at the same dose (Table 4). However, the opposite trend was observed for EC, which decreased significantly with the increased rate of vermicompost application, regardless of the combined sowing methods and seedbed types (Table 4).

**Table 4:** Residual effect of vermicompost and cropping systems on soil EC and CEC in 2022 years at Kile.

Vermicompost (t/ha)	Electric conductivity (dS/cm)				Cation exchange capacity (cmol (+)/Kg soil)			
	Intercrop	Monocrop	F	TD	Intercrop	Monocrop	F	TD
0	63.517^a^	61.813^a^	67.94^a^	57.39^b^	15.092^e^	16.337^e^	15.31^e^	16.12^e^
1.5	54.077^b^	45.637^c^	52.73^c^	46.98^c^	19.815^d^	20.167^d^	20^d^	19.98^d^
3	32.933^d^	31.883^de^	33.47^e^	31.35^ef^	23.398^c^	22.822^c^	23.38^c^	22.84^c^
4.5	30.063^de^	29.53^e^	29.96^f^	29.64^f^	33.362^a^	26.223^b^	31.62^a^	27.97^b^
LSD	3.004				2.332			

TD =  tied-ridge; F = open-furrow (traditional practice); LSD =  least significant difference. Means sharing the same letters under each variable was non-significant.

#### Total nitrogen (N) and available phosphorus (P).

The results in Table 3 showed that total nitrogen and available Olsen P were significantly (*P* < 0.05) influenced by the two-way interaction and main effects of vermicompost and cropping systems. However, seedbed types, either individually or in interaction with both factors, did not significantly affect soil N and P (Table 3). Treatments previously amended with 4.5 t/ha of vermicompost under the sorghum-groundnut intercrop system resulted in the highest total N (0.242%) and available P (9.822 mg/Kg) (Table 5). Whereas the control plot (unamended soil) gave the lowest total N (0.007%) and Olsen P (3.57 mg/Kg). Compared to solely cropped sorghum at nil fertilizer, the previous intercropped sorghum-groundnut system increased soil N and P by about 8.69% and 4.75%, respectively, when it was treated with 4.5 t/ha vermicompost (Table 5). Except for the plot that received the highest vermicompost rate, all plots that received the same rate in both intercropping and monocropped systems were statistically insignificant from each other for N and P (Table 5).

**Table 5:** Residual effect of vermicompost and cropping systems on soil N and P in 2022 years at Kile.

Vermicompost (t/ha)	Total Nitrogen (N%)				Available Phosphorus (Olsen P mg/Kg)			
	Intercrop	Monocrop	F	TD	Intercrop	Monocrop	F	TD
0	0.008^ef^	0.007^f^	0.008^e^	0.009^e^	3.868^e^	3.573^e^	3.748^f^	3.693^f^
1.5	0.093^de^	0.099^d^	0.094^d^	0.098^d^	4.762^d^	4.582^d^	4.727^e^	4.617^e^
3	0.135^c^	0.125^c^	0.13^c^	0.13^c^	7.04^c^	6.935^c^	7.457^c^	6.518^d^
4.5	0.247^a^	0.208^b^	0.242^a^	0.213^b^	9.822^a^	8.93^b^	9.677^a^	9.075^b^
LSD	0.014				0.495			

TD =  tied-ridge; F =  open-furrow planting pattern; LSD =  least significant difference. Means sharing the same letters under each variable were non-significant.

#### Exchangeable bases (K, Ca, and Mg).

**Fig 4:**
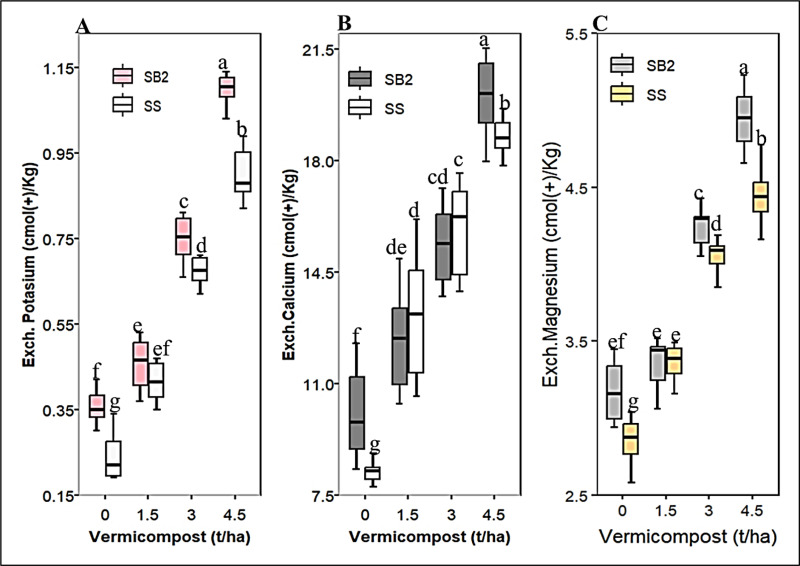
Two-way interaction effect of vermicompost and planting methods on soil Exch. Potassium (A), Exch. calcium (B), and Exch. Magnesium (C). Box sharing the same letter under each figure was statistically similar.

Single and joint applications of vermicompost and sowing methods were significant (*P* < 0.05) for exchangeable bases (S4 Table in S1 File). However, exchangeable Ca was not significantly affected by a single effect of cropping and seedbed types, unlike other bases (Table 3). The highest values for all exchangeable bases were obtained in plots treated with 4.5 t/ha vermicompost under an intercropping scheme (Fig 4). The study revealed that the minimum exchangeable bases in sorghum grown in monocultures without vermicompost and intercropped with nil fertilizer were statistically identical to those in plots receiving 1.5 t/ha of vermicompost. In the intercropping system at 4.5 t/ha vermicompost, the concentration of exchangeable K, Mg, and Ca increased linearly by 64.18%, 25%, and 37.31%, respectively (Fig 4A–C).

#### Extractable zinc (Zn), iron (Fe), and manganese (Mn).

The combined application of vermicompost with different sowing methods had a significant residual effect on extractable micronutrients (Fe, Mn, and Zn) (Table 3). The mean effect of all three factors was significant for Zn, while for Mn and Fe, only the single effects of sowing methods and vermicompost showed significant differences. Increasing rates of vermicompost under intercropping increased Zn and Fe by 40.1% and 27.15%, respectively (Table 6). However, Mn decreased by 2.56% with increased vermicompost rates. The lowest Mn (1.97 mg/Kg soil) was found at 4.5 t/ha in monocropped sorghum, which was statistically similar to the plot that received 1.5 t/ha. On the other hand, the highest mean values of extractable Fe (6.37 mg/Kg) and Zn (1.45 mg/Kg) were recorded at 4.5 t/ha of vermicompost under the intercropping system (Table 6).

**Table 6:** Interaction effect of vermicompost and sowing methods on Zn, Fe, and Mn in 2022 years at Kile.

Vermicompost (t/ha)	Zn (mg/Kg)		Fe (mg/Kg)		Mn (mg/Kg)	
	Intercrop	Monocrop	Intercrop	Monocrop	Intercrop	Monocrop
0	0.62^f^	0.28^g^	3.65^f^	3.73^f^	2.01^bcd^	2.47^a^
1.5	0.86^d^	0.74^e^	4.17^e^	4.22^e^	1.98^cd^	2.16^b^
3	1.18^b^	1.08^c^	5.16^c^	4.72^d^	1.89^d^	2.08^bc^
4.5	1.45^a^	1.18^b^	6.37^a^	5.7^b^	1.90^d^	1.97^cd^
LSD	**0.08**		**0.38**		**0.18**	

LSD =  least significant difference; Zn =  zinc; Fe =  iron; Mn =  manganese. Means sharing the same letters under each variable were non-significant.

#### Soil moisture content (SMC).

The ANOVA results in Table 3 indicate a significant difference (*P* < 0.05) among all treatment combinations in SMC during the 2022 cropping season. The highest SMC levels (>26.72%) at both stages were observed in tied-ridging under the intercropping system (Table 6). Specifically, the maximum SMC under tied-ridging at 4.5 t/ha exceeded the control plot by 47% and 46% at the vegetative and reproductive stages, respectively. Conversely, the lowest SMCs were found in both sole sorghum and previously intercropped sorghum without fertilizer (Table 7). Tied-ridge planting increased SOM by 16.13% in previously groundnut-intercropped fields at the vegetative stage and by 16.67% in sole cropping of sorghum at the reproductive stage without fertilizer application (Table 7). The SMC recorded at the early flowering stage generally exhibited a declining trend, which was more pronounced in treatments employing openfurrow (Table 7), likely due to the typically reduced rainfall during the critical reproductive stage (mid-September-November) in the study area (Fig 1).

**Table 7:** Interaction effect of vermicompost, seedbed type, and cropping systems on soil moisture content (SOM) during the 2022 year at Kile.

Treatments	Soil moisture (%) at vegetative stage				Soil moisture (%) at reproductive stage			
Residual VC (t/ha)	0	1.5	3	4.5	0	1.5	3	4.5
SSF	**12.99** ^**h**^	15.45^g^	18.16^ef^	20.23^cde^	**9.99** ^**k**^	12.76^ij^	14.91^fgh^	17.44^cde^
SSTd	16.22^fg^	18.36^ef^	20.65^cd^	26.99^b^	14.05^ghi^	15.12^fg^	16.06^ef^	19.57^b^
SS1F	**13.11** ^**h**^	15.61^g^	18.01^ef^	19.19^de^	**11.21** ^**kj**^	13.24^hi^	16.16^def^	17.96^bcd^
SS1Td	18.15^ef^	21.89^c^	28.39^b^	**35.75** ^**a**^	13.96^ghi^	15.05^fgh^	18.51^bc^	**26.72** ^**a**^
LSD	2.64				1.84			

SSF/Td = sole sorghum under present F = open-furrow; Td =  tied ridge; SS1F/Td = previous intercropped sorghum with *Babile-1* under open-furrow (F) and tied ridge (Td), respectively; VC =  vermicompost; LSD = least significant difference. Mean sharing the same letter are insignificantly different.

#### Residual effect of vermicompost and groundnut on *Striga* density.

**Fig 5:**
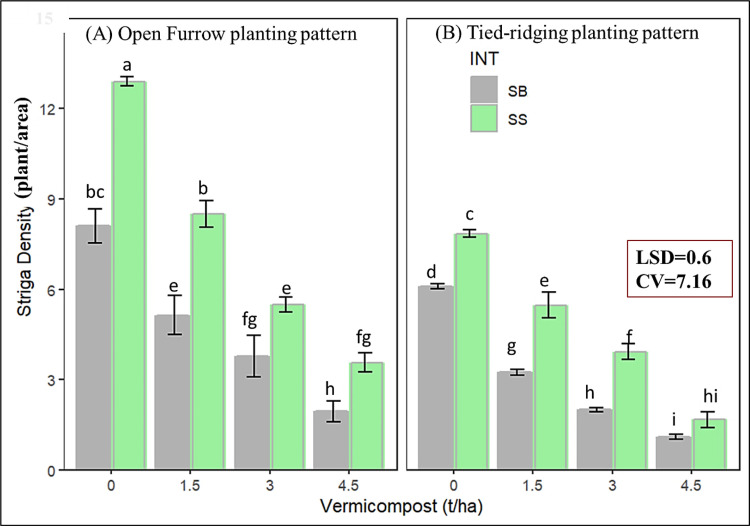
Three-way interaction effect of all factor on *Striga* density; LSD=Least significant difference; CV=Coefficient of variation. Bar sharing the same letter under each figure were statistically similar.

The ANOVA result in Table 3 depicts the main effect as well as the combination effect of all factors significantly (*P* ≤ 0.01) affecting *Striga* density (S5 Table in S1 File). The highest *Striga* density (12.9 plants/plot) was observed in monocropped sorghum under the traditional tillage system and was significantly different from the other treatments (Fig 5). The minimum *Striga* density (1.09 plants/plot) was recorded at 4.5 t/ha of residual vermicompost applied under tied-ridging in the previous intercropping system, at par with sole-cropped sorghum at the same treatment combinations. Increased vermicompost from nil to 4.5 t/ha declines *Striga* density by about 64 and 71% in monocropped and intercropped sorghum (Fig 5).

#### Sorghum stover yield and grain yield.

Residual vermicompost under both cropping systems substantially affects both yield parameters of sorghum (Table 2). Accordingly, the highest stover yield (10.95 t/ha) was recorded under tied-ridge with *Babile-1* at 4.5 t/ha vermicompost, while the lowest sorghum grain yield (3.213 t/ha) was attained under control (sole cropped without fertilizer) (Table 8a). In comparison to the control plot (SSF), the application of vermicompost at 4.5 t/ha under previous intercropped groundnut boosted stover yield by 54% (Table 8a). Likewise, the greatest sorghum grain yield (5.817 t/ha) recorded at 4.5 t/ha residual vermicompost with a tied-ridge planting pattern was nearly three times more than the control plot (Table 8b). Compared to pure-stand sorghum without fertilizer, residual vermicompost application at 4.5 t/ha in an intercropping system enhanced grain yield by 57.47% (Table 8b).

**Table 8:** *Residual* effect of vermicompost under previous intercropping and both seedbed types on sorghum stover yield and grain yield at Kile during the 2022 year.

Cropping pattern	(a) Stover yield (t/ha)					(b) Grain yield (t/ha)			
	Residual VC (t/ha)	0	1.5	3	4.5	0	1.5	3	4.5
SSF		3.213^e^	3.9474^e^	5.804^d^	8.073^b^	1.445^f^	2.538^e^	3.264^d^	4.162^c^
SSTd		3.766^e^	6.018^cd^	8.206^bc^	8.206^b^	1.627^f^	2.436^e^	3.839^c^	5.285^a^
SS1F		3.49^e^	5.28^d^	6.852^c^	8.339^b^	1.503^f^	2.49^e^	3.236^d^	4.782^b^
SS1Td		3.59^e^	5.49^d^	8.178^b^	10.81^a^	2.291^e^	2.728^e^	4.68^b^	5.35^a^
LSD		**1.0**				**0.082**			

SSF/Td = sole sorghum under present F = open-furrow; Td =  tied ridge; SS1F/Td = previous intercropped sorghum with Babile-1 under open-furrow (F) and tied ridge (Td), respectively; VC =  vermicompost; LSD = least significant difference. Mean sharing the same letter are insignificant.

## Discussion

### Properties of vermicompost and soil before experiment

The high concentration of organic matter in vermicompost may be attributed to the abundance of biomass found in *Lantana camara* and animal feces, which act as substrates. The higher EC of vermicompost compared to pre-sowing soil is most likely due to increased soluble salts in vermicompost following worm activity [36]. Vermicompost has the optimum nutrient C:N ratio and high-status accessible nutrients used for plant growth [20,77]. This high OM and CEC content in vermicompost was most likely responsible for the higher levels of macro- and micronutrients [21,36].

The soil in the research area has a sandy loam texture with 66.78% sand and 15.06% clay content, making it ideal for sorghum and groundnut production. However, according to Wogi *et al*. [78], the soils in the research area are extremely deficient in soil N, P, K, SOM, and other nutrients. Similarly, the maximum soil bulk density (1.45 g/cm^3^) in Kile may be attributable to excessive compaction from extensive sorghum monocropping and constant tractor use. These issues highlight the need for using high-quality organic fertilizer (vermicompost), which has a significantly higher concentration of SOM and most importantly plant nutrients than soil. This could rectify the plant nutrient imbalance and increase productivity without endangering the agroecosystem [78,79].

### Residual effect of vermicompost and groundnut on selected soil physico-chemical characteristics in sorghum field at Kile in 2022 year

#### Soil organic carbon (SOC), bulk density (BD), and moisture content (SMC).

Vermicompost is an excellent soil conditioner that can aid in environmentally friendly farming practices. The SMC increased in plots with tied-ridging, groundnut intercropping, and vermicompost treatment, possibly due to lower bulk density and higher SOC levels from previous years. Bekele & Chemeda [80] found that tied ridging increased soil water and sorghum grain yield by more than 25% and 40%, respectively, compared to regular tillage in northern Ethiopia. Abie *et al*. [81] observed that plots treated with tied ridges had higher soil moisture content than the control plot. In addition, the organic matter in vermicompost binds soil particles together to form stable, larger aggregates with holes, lowering bulk density and improving water retention by slowing surface water flow [64]. The lower soil BD and linear increase in SOC are most likely due to a higher fraction of SOM return from groundnut biomass [82]. Similarly, a large surface area and low C/N ratios of vermicompost speed up decomposition, boosting SOM while decreasing soil bulk density [83]. In sandy soils, high SOM and low BD increase soil water-holding capacity, besides improving soil physicochemical features [24]. Bhanwaria *et al*. [20] also reported that applying 5 t/ha of vermicompost increased soil moisture retention and accessible soil water in mustard crops under stress in arid environments. Bhanwaria *et al*. [20]; Hafez *et al*. [50]; and Demir [84] additionally endorsed our findings, stating that soil organic matter is a significant source of accessible nutrients, soil water-holding capacity, and CEC that maintain soil stability. According to research by Angelova *et al*. [85], adding 5 and 10 grams of vermicompost to soil boosted its organic content by 3.99% and 9.36%, respectively. According to González *et al*. [18], vermicompost applied once at a dose of 2 kg m^-2^ was enough to significantly lower the bulk density of the soil. Atsbha *et al*. [86] and Li *et al*. [87] conveyed comparable findings.

Our research findings about intercropping also aligned with those of Roohi *et al*. [6], Talababie *et al*. [88], and Nasar *et al*. [89], who found that intercropped maize with high biomass and litter production had the maximum amount of soil organic matter. Attallah *et al*. [90] found that when compared to monocropping, intercropping durum wheat with chickpeas increased soil carbon levels. A higher SOC results from microbial activity in the soil breaking down vermicompost and residual intercropping, which promotes soil fertility and carbon sequestration [6,91]. Increased groundnut and sorghum litter results in a larger SOM stock, which increases the amount of labile carbon [56]. This illustrates the necessity of using tied-ridge planting patterns and vermicompost to maintain optimal SMC levels, improve soil fertility, and reduce irrigation frequency.

#### Soil pH.

The slight increase in soil pH in residual vermicompost could be attributed to the high pH and basic cations contained in vermicompost [92,93]. However, the increased release of H^ +^ ions from groundnuts caused by N_2_-fixation, excessive cation absorption, and root exudation of organic acids significantly reduces soil pH in the intercropping system [94,95]. This proves that the combined application of vermicompost and intercropping systems aids in buffering soil pH within the range that is optimal for crop growth and the availability of plant nutrients [85,96–98]. Additionally, vermicompost can neutralize excessive acidity or alkalinity in soil, hence buffering its pH [90]. This finding is consistent with Bhanwaria *et al*. [20] and Adebayo *et al*. [92], who revealed that residual vermicompost raised soil pH, SOC, P, and K levels. According to Wegene *et al*. [98], the use of vermicompost increased soil pH from 5.1 to 5.5 due to humic chemicals produced during H^ + ^-consuming decomposition processes. Beshir & Abdulkerims [9] noted that the pH of the soil was lower when the intercropping was done with a closed-end tied ridge. The observed lower soil pH in the tied-ridge planting pattern may be due to proton generation linked to nitrification, as lower redox potential is generally reported in saturated or wet soil [99]. Nasar *et al*. [89]; Guerchi *et al*. [95]; and Saha *et al*. [100] found similar results in cereal-legume intercropping.

#### Cation exchange capacity (CEC) and electric conductivity (EC).

The results of the study indicate that the addition of vermicompost and groundnut intercropping has a considerable impact on the soil CEC and EC, probably due to an increase in soil OM content. Increased soil organic matter and the formation of additional phenolic and carboxylic functional groups could explain the rise in CEC [101,102]. High CEC increases nutrient availability for plant development by reducing leaching [103,104]. The soil EC may have decreased due to vermicompost’s high sorption capacity, which allows it to absorb a large amount of salts and amino acids created during microbial decomposition [93,105]. Similar outcomes were found by Bhanwaria *et al*. [20], who reported that a decrease in soil pH and EC led to a decrease in soil salinity and an improvement in soil quality. According to Guerchi *et al*. [95], intercropping sea and alfalfa hardly lowers soil salinity in arid and semi-arid areas, which supports sustainable farming practices.

#### Total nitrogen (N) and available phosphorus (P).

The highest residual soil N and P levels in the second year are most likely caused by a high rate of higher total organic C and N return from vermicompost as well as greater groundnut leaf drop along with a significant amount of fixed nitrogen throughout the growth season [29]. Soils treated with vermicompost were more likely to resist nutrient immobilization and retain more accessible N and P at the end of the growth cycle due to the greater availability of organic matter [91]. Additionally, vermicompost reduces the P fixation and enhances the P mobilization via its rich organic acids, which compete for binding sites with orthophosphates and complexes with metals, thereby slowing down the formation of sparingly soluble phosphates [97]. The current finding has been supported by Jat & Ahlawat [25], Angelova *et al*. [85], and Mengistu *et al*. [106], who reported that vermicompost outperformed the control treatment in terms of available and total N. Similar to this, Talababie *et al*. [88] discovered a rise in soil P and N levels during the first season of a legume-cereal intercropping system.

Intercropping improved the microclimate, biomass returned to the soil (exudates, root debris, and leaf shade), and soil microbial activity [58], all of which accelerated the breakdown of resistant carbon such as cellulose and lignin [54] soil‑based N, P, K, NH_4_, NO_3_, and SOC [89]. According to the Ncube *et al*. [107] study, groundnut and cowpea legumes accumulated up to 130 kg of nitrogen per hectare, which sorghum may absorb in the following season. Intercropped groundnut fixed the highest amount of nitrogen from the atmosphere and transferred 12–26% more nitrogen to base crops than cowpea (*Vigna unguiculata* L.) and mungbean (*Vigna radiata* L.) [108].

The residual Olsen P in the second year at 4.5 t/ha, vermicompost exhibits the lowest range, demonstrating definitely the need for additional P fertilizer for the crop the following year. This could have been brought on by microbial immobilization [61] high adsorption of P to leftover Ca, Mg, and K [109] or significant P removal from the soil through harvested materials. However, the residual effect of total N in vermicompost was classified as high to very high at 3 and 4.5 t/ha [78]. It would be able to gain the benefits of large doses of vermicompost for more than two successive cropping. When cassava and peanuts were interplanted for three years in a row, accessible N, P, K, and organic matter rose by 40% and nearly 20 times, respectively, compared to the control soils [110].

#### Exchangeable bases (K, Ca, and Mg).

The greatest number of exchangeable bases identified at high vermicompost dosages were linked to high CEC, which had a significant buffering capacity and the ability to hold cations against leaching [106,111]. These findings are consistent with those of Demir [84], who discovered that raising the vermicompost rate significantly increased the exchangeable base K, Mg, and Ca levels in the soil. Vermicompost increases potassium, exchangeable magnesium, accessible phosphorus, and SOM [112]. Organic amendments increased soil exchangeable K, Mg, Ca, and CEC [113]. Maize-haricot bean intercropping improved the likely availability of potassium in the soil after planting [9]. The soil exchangeable Ca and Mg levels ranged from high to extremely high, according to the Wogi *et al*. [78] rating. This shows that additional soil inputs are unnecessary because the agricultural soils in this area do not suffer from calcium and magnesium deficits. However, residual exchangeable K decreased, necessitating further K-containing fertilizer inputs. Intercropping improved soil physicochemical properties by boosting ecosystem functioning, such as soil microbial biomass, enzyme activity, and nutrient cycling [58].

#### Extractable zinc (Zn), iron (Fe), and manganese (Mn).

Vermicompost often contains more nutrients than conventional compost produced from the same substrate because earthworms digest and fragment the material in biochemical form, adding vitamins, antibiotics, enzymes, and growth hormones [96]. Increased soil organic carbon and pH were most likely accountable for the highest extractable Zn and Fe levels in the previously treated plot with high vermicompost dosage. Furthermore, the application of vermicompost could stimulate the growth of the Fe-reducing microorganisms, which have a synergistic effect with OM, thereby enhancing Fe reduction [97]. These findings were consistent with Demir [84], who reported an increasing trend in the micronutrients (Fe, Mn, Zn, and Cu) in vermicompost with different watering regimens. The macronutrient and micronutrient content of the postharvest soil within the cauliflower field increased as vermicompost application doses increased [114]. Mengistu *et al*. [106] found that the high organic matter content of vermicompost enhances micronutrient concentrations (Fe, Mn, Zn, and Cu) in soil through oxidation and decomposition. Nevertheless, our results show that extractable Mn levels decreased as vermicompost levels increased. This could be linked to a rise in soil pH, which could lead to the development of low-solubility compounds and better soil colloidal retention [115]. Mn shortages caused by high vermicompost rates have been connected to low Mn and high SOM, which firmly contained it [116]. In contrast, because Zn is not strongly linked to organic materials, its availability increases.

### Residual effect of vermicompost on *Striga* density and associated sorghum yield

#### *Striga* density.

The decline in *Striga* density under the highest fertilizer dose could be linked to the lower C:N ratio as well as the high NPK and SOM content of vermicompost [86]. Furthermore, the addition of vermicompost significantly improved post-harvest soil microbial activity and important nutrients [21,117]. Another aspect could be improved availability of minerals such as zinc, boron, and magnesium, which help to create mechanical barriers (lignification or phenolic compounds) and hence improve host-plant resilience in the post-attachment stages [16,17]. The result aligned with the findings of Gacheru & Rao [118], who discovered that organic residues can reduce *Striga* seed germination and viability. Goldwasser & Rodenburg [119] discovered that the breakdown of organic components in an intercropping system results in nutrient release, enhanced biological activity, and the generation of ethylene gas, causing *Striga* to germinate. *Striga* emergence, attachments, and germination were reduced when the host plant reduced strigolactone exudation [120,121].

Furthermore, vermicompost decomposition [22,32] resulted in plant growth regulators, increased microbial populations and activities [69], and increased host-plant resistance to pest attacks [91]. Likewise, Woldemariam & Damot [122] explored the management of animal manure and nitrogen. Restore biomass productivity to rejuvenate and rebuild soil. Under legume cultivation, soil organic carbon content increased and the number of *Striga* seeds per square meter decreased (Abunyewa & Padi, 2003) [123]. Tesso & Ejeta [14] noted that tied-ridge tillage reduced the number of emerged *Striga* by two to three times at the last scoring dates as well as significantly lowering *Striga* vigor. Cereal-legume intercropping also shelters the soil from direct sunshine, reducing evaporation and increasing soil water retention for plant uptake [9].

#### Sorghum yield.

The findings revealed that residual vermicompost at 4.5 t/ha yielded the highest sorghum yield, most likely due to significant nutrient storage and reservation at this rate. Improved nutrient availability and soil structure influenced plant vegetative development and yield characteristics [124]. According to a meta-analysis of Mak-Mensah *et al*. [125] and a finding of Mesfin *et al*. [74], the tied-ridge planting pattern had a yield advantage over flat planting for sorghum of more than 17%. Tie-ridging may boost sorghum grain output by making better use of the retained soil moisture, soil organic carbon, and total nitrogen. Tied-ridging may boost sorghum grain yield by making better use of the retained soil moisture, soil organic carbon, and total nitrogen. Adebayo *et al*. [92] reported similar results that residual vermicompost boosted cucumber (*Cucumis sativus* L.) production in the following plantings. Furthermore, by enhancing N, P, and K uptake, the residual application of farmyard manure plus 75% NPK increased wheat grain yield considerably [124]. Dass *et al*. [126] revealed that the cumulative influence of earlier black-gram intercropping significantly increased the growth and productivity of the current wheat crop. Peanut and maize intercropping affected the composition of soil microorganisms that aid in plant development and nutrient uptake [127]. Hence, such integrated practice could provide a comprehensive way to produce sorghum while improving soil fertility and reducing *Striga* infestation.

## Conclusion

The results revealed that soil organic carbon (SOC), nitrogen (N), calcium (Ca), magnesium (Mg), zinc (Zn), and iron (Fe) levels linearly increased with the application rate of vermicompost under an intercropping system. The soil moisture content (SMC) also had a significant increase in both sorghum growth stages due to the residual effect of vermicompost under a tied-ridge planting pattern. The combined application of high vermicompost doe and tied-ridge in preceding intercropped groundnut gave the highest sorghum yields by decreasing *Striga* infestation. Hence, preceding groundnut intercropping and residual vermicompost not only improve soil fertility and moisture retention but also reduce *Striga* density, thereby offering promising prospects for sustainable sorghum production in the semi-arid regions of the eastern Hararghe zone. The aforementioned treatment showed a slight decline in soil pH, electrical conductivity (EC), and bulk density (BD), as well as available phosphorus (P) and potassium (K), compared to the initial soil analysis result but higher than the current control plot. This indicates depletion of P and K after the second planting, which suggests residual effects of vermicompost were sufficient for one planting cycle for P and K. However, other soil nutrients left in the soil system after the second cropping season ranged between medium and high at 4.5 t/ha of vermicompost, henceforth might be available for the third cropping cycle. Similarly, monocropped sorghum without fertilizer exhibited the lowest levels of macro- and micronutrients, attributed to the intensive nutrient uptake by sorghum over two years. This greatly benefits smallholder farmers in terms of economic return, ecological safety, and nutritional quality, particularly from groundnut.

## Supporting information

S1 File S1 Table. Soil pH as influenced by Vermicompost with cropping systems [seedbed types (A) and sowing methods (B)]. S2 Table. Soil organic carbon (SOC) as influenced by Vermicompost with sowing methods. S3 Table. Potassium (K) as influenced by Vermicompost with sowing methods. S4 Table. Soil Calcium (Ca) as influenced by Vermicompost with sowing methods. S5 Table. Soil Magnesium (Mg) as influenced by Vermicompost with sowing methods. S6 Table. Striga density as influenced by Vermicompost with cropping systems.
(DOCX)

## References

[1] Bekunda M, Sanginga N, Woomer PL. Restoring soil fertility in sub-Sahara Africa. Adv Agron. 2010;108:183–236. doi: 10.1016/s0065-2113(10)08004-1

[2] Tully KL, McAskill C. Promoting soil health in organically managed systems: a review. Org Agr. 2019;10(3):339–58. doi: 10.1007/s13165-019-00275-1

[3] Tonitto C, Ricker-Gilbert JE. Nutrient management in African sorghum cropping systems: applying meta-analysis to assess yield and profitability. Agron Sustain Dev. 2016;36(1). doi: 10.1007/s13593-015-0336-8

[4] Erkossa T, Laekemariam F, Abera W, Tamene L. Evolution of soil fertility research and development in Ethiopia: from reconnaissance to data-mining approaches. Ex Agric. 2022;58. doi: 10.1017/s0014479721000235

[5] Kifle D. Soil fertility management and cropping system function in ameliorating maize productivity in Ethiopia. World J Agri soil Sci. 2020;4(2):1–11. doi: 10.33552/WJASS.2020.04.00058

[6] Roohi M, Saleem Arif M, Guillaume T, Yasmeen T, Riaz M, Shakoor A, et al. Role of fertilization regime on soil carbon sequestration and crop yield in a maize-cowpea intercropping system on low fertility soils. Geoderma. 2022;428:116152. doi: 10.1016/j.geoderma.2022.116152

[7] Esilaba A O, Reda F, Ransom JK, Bayu W, Woldewahid G, Zemichael B. Integrated nutrient management strategies for soil fertility improvement and Striga control in northern Ethiopia. African Crop Sci J. 2000;8(4). doi: 10.4314/acsj.v8i4.27680

[8] Tadele Z. Raising crop productivity in Africa through intensification. Agronomy. 2017;7(1):22. doi: 10.3390/agronomy7010022

[9] Beshir S, Abdulkerim J. Effect of maize/haricot bean intercropping on soil fertility improvement under different tied ridges and planting methods, southeast Ethiopia. GEP. 2017;05(08):63–70. doi: 10.4236/gep.2017.58007

[10] Yoshida S, Shirasu K. Multiple layers of incompatibility to the parasitic witchweed, *Striga hermonthica*. New Phytologist. 2009;183(1):180–9. doi: 10.1111/j.1469-8137.2009.02840.x 19402875

[11] David OG, Ayangbenro AS, Odhiambo JJO, Babalola OO. *Striga hermonthica*: a highly destructive pathogen in maize production. Environ Challenges. 2022;8:100590. doi: 10.1016/j.envc.2022.100590

[12] Kroschel J. A technical manual for parasitic weed research and extension. 2001. doi: 10.1007/978-94-010-0005-5

[13] Kokla A, Leso M, Zhang X, Simura J, Serivichyaswat PT, Cui S, et al. Nitrogen represses haustoria formation through abscisic acid in the parasitic plant *Phtheirospermum japonicum*. Nat Commun. 2022;13(1):2976. doi: 10.1038/s41467-022-30550-x 35624089 PMC9142502

[14] Tesso TT, Ejeta G. Integrating multiple control options enhances Striga management and sorghum yield on heavily infested soils. Agronomy J. 2011;103(5):1464–71. doi: 10.2134/agronj2011.0059

[15] Kamara AY, Ekeleme F, Jibrin JM, Tarawali G, Tofa I. Assessment of level, extent and factors influencing Striga infestation of cereals and cowpea in a Sudan Savanna ecology of northern Nigeria. Agric Ecosyst Environ. 2014;188:111–21. doi: 10.1016/j.agee.2014.02.027

[16] Mwangangi IM, Büchi L, Haefele SM, Bastiaans L, Runo S, Rodenburg J. Combining host plant defence with targeted nutrition: key to durable control of hemiparasitic Striga in cereals in sub-Saharan Africa?. New Phytol. 2021;230(6):2164–78. doi: 10.1111/nph.17271 33577098

[17] Mwangangi IM, Büchi L, Runo S, Rodenburg J. Essential plant nutrients impair post‐germination development of Striga in sorghum. Plants People Planet. 2023;7(2):422–35. doi: 10.1002/ppp3.10418

[18] González M, Gomez E, Comese R, Quesada M, Conti M. Influence of organic amendments on soil quality potential indicators in an urban horticultural system. Bioresour Technol. 2010;101(22):8897–901. doi: 10.1016/j.biortech.2010.06.095 20630748

[19] Nord A, Snapp S, Traore B. Current knowledge on practices targeting soil fertility and agricultural land rehabilitation in the Sahel. A review. Agron Sustain Dev. 2022;42(4). doi: 10.1007/s13593-022-00808-1

[20] Bhanwaria R, Singh B, Musarella CM. Effect of organic manure and moisture regimes on soil physiochemical properties, microbial biomass Cmic:Nmic:Pmic turnover and yield of mustard grains in arid climate. Plants (Basel). 2022;11(6):722. doi: 10.3390/plants11060722 35336604 PMC8949599

[21] Oyege I, Balaji Bhaskar MS. Effects of vermicompost on soil and plant health and promoting sustainable agriculture. Soil Systems. 2023;7(4):101. doi: 10.3390/soilsystems7040101

[22] Mutsvanga S, Gasura E, Setimela PS, Nyakurwa CS, Mabasa S. Nutritional management and maize variety combination effectively control Striga asiatica in southern Africa. CABI Agric Biosci. 2022;3(1). doi: 10.1186/s43170-022-00108-4

[23] Muktamar T, Adiprasetyo S, Yulia L, Sari F, Fahrurrozi F, Setyowati N. Residual effect of vermicompost on sweet corn growth and selected chemical properties of soils from different organic farming practices. Inter J Agric Techn. 2018;14(7):1471–82.

[24] Bouajila K, Hechmi S, Mechri M, Jeddi FB, Jedidi N. Short-term effects of Sulla residues and farmyard manure amendments on soil properties: cation exchange capacity (CEC), base cations (BC), and percentage base saturation (PBS). Arab J Geosci. 2023;16(7). doi: 10.1007/s12517-023-11487-x

[25] Jat RS, Ahlawat IPS. Direct and residual effect of vermicompost, biofertilizers and phosphorus on soil nutrient dynamics and productivity of chickpea-fodder maize sequence. Journal of Sustainable Agriculture. 2006;28(1):41–54. doi: 10.1300/j064v28n01_05

[26] Dhaliwal SS, Sharma V, Shukla AK, Verma V, Kaur M, Singh P, et al. Effect of addition of organic manures on basmati yield, nutrient content and soil fertility status in north-western India. Heliyon. 2023;9(3):e14514. doi: 10.1016/j.heliyon.2023.e14514 36967980 PMC10031471

[27] Mugwe J, Otieno EO. Integrated Soil Fertility Management Approaches for Climate Change Adaptation, Mitigation, and Enhanced Crop Productivity. In: Handbook of Climate Change Management. Leal Filho W, editor. Switzerland AG: Springer Nature; 2021, p. 1–22.

[28] Makkar C, Singh J, Parkash C, Singh S, Vig AP, Dhaliwal SS. Vermicompost acts as bio-modulator for plants under stress and non-stress conditions. Environ Dev Sustain. 2022;25(3):2006–57. doi: 10.1007/s10668-022-02132-w

[29] Stomph T. Designing intercrops for high yield, yield stability and efficient use of resources: Are there principles? 1st ed., vol. 160, no. 1. Elsevier Inc.; 2020.

[30] Bidabadi SS, Dehghanipoodeh S, Wright GC. Vermicompost leachate reduces some negative effects of salt stress in pomegranate. Int J Recycl Org Waste Agricult. 2017;6(3):255–63. doi: 10.1007/s40093-017-0173-7

[31] Ma H, Zhao S, Hou J, Feyissa T, Duan Z, Pan Z, et al. Vermicompost improves physicochemical properties of growing medium and promotes plant growth: a meta-analysis. J Soil Sci Plant Nutr. 2022;22(3):3745–55. doi: 10.1007/s42729-022-00924-7

[32] ur Rehman S, De Castro F, Aprile A, Benedetti M, Fanizzi FP. Vermicompost: enhancing plant growth and combating abiotic and biotic stress. Agronomy. 2023;13(1134):1–25. doi: 10.3390/agronomy13041134

[33] Taneja T, Sharma I, Singh BJ, Singh A, Kumar M, Singh R. Composting as a sustainable option for converting undesirable weeds like *Parthenium hysteropherous*, *Solanum nigrum*, *Calotropis procera* and *Trianthema portulacastrum* into organic manure. Biosci Biotech Res Asia. 2024;21(2):645–54. doi: 10.13005/bbra/3253

[34] Devi R, Kumar A, Deboch B. Organic farming and sustainable development in Ethiopia. Sci Res Essay. 2007;2(6):199–203.

[35] Mekonnen E, Argaw A. Bioconversion of Wastes (Khat Leaf Leftovers and Eucalyptus Twigs) into Vermicompost and Assessing Its Impact on Potato Yield. J Agron. 2014;14(1):37–42. doi: 10.3923/ja.2015.37.42

[36] Yadav A, Garg VK. Vermicomposting--An effective tool for the management of invasive weed *Parthenium hysterophorus*. Bioresour Technol. 2011;102(10):5891–5. doi: 10.1016/j.biortech.2011.02.062 21392980

[37] Hussain N, Abbasi T, Abbasi SA. Vermicomposting transforms allelopathic parthenium into a benign organic fertilizer. J Environ Manage. 2016;180:180–9. doi: 10.1016/j.jenvman.2016.05.013 27233043

[38] Rameshwar HY, Argaw A. Manurial value of khat waste vermicompost from Awday, Harar town, Ethiopia. Int J Recycl Org Waste Agricult. 2016;5(2):105–11. doi: 10.1007/s40093-016-0121-y

[39] Suthar S, Sharma P. Vermicomposting of toxic weed--*Lantana camara* biomass: chemical and microbial properties changes and assessment of toxicity of end product using seed bioassay. Ecotoxicol Environ Saf. 2013;95:179–87. doi: 10.1016/j.ecoenv.2013.05.034 23796668

[40] Sharma K, Garg VK. Conversion of a toxic weed into vermicompost by Eisenia fetida: Nutrient content and earthworm fecundity. Bioresource Technology Reports. 2020;11:100530. doi: 10.1016/j.biteb.2020.100530

[41] Singh A, Singh GS. Vermicomposting: a sustainable tool for environmental equilibria. Environmental Quality Mgmt. 2017;27(1):23–40. doi: 10.1002/tqem.21509

[42] Gebrehana ZG, Gebremikael MT, Beyene S, Wesemael WML, De Neve S. Assessment of trade-offs, quantity, and biochemical composition of organic materials and farmer’s perception towards vermicompost production in smallholder farms of Ethiopia. J Mater Cycles Waste Manag. 2022;24(2):540–52. doi: 10.1007/s10163-021-01339-9

[43] Sintayehu D, Warkineh B. Current status and future invasion potential of *Lantana camara* L. under the changing climate and land cover in the Central Rift Valley, Ethiopia. Ethiopian Journal of Biological Sciences. 2020;19(1):103–16.

[44] Qin Z, Zhang JE, DiTommaso A, Wang RL, Liang KM. Predicting the potential distribution of *Lantana camara* L. under RCP scenarios using ISI-MIP models. Climatic Change. 2015;134(1–2):193–208. doi: 10.1007/s10584-015-1500-5

[45] Bobo A, Abdeta C. A review on the distribution, biology and management practices of parthenium weed, (*Parthenium hysterophorus* L.) in Ethiopia. J Biol Agric Heal. 2016;6(5):136–45.

[46] Mcconnachie AJ, Strathie LW, Mersie W, Gebrehiwot L, Zewdie K, Abdurehim A, et al. Current and potential geographical distribution of the invasive plant *Parthenium hysterophorus* (Asteraceae) in eastern and southern Africa. Weed Research. 2010;51(1):71–84. doi: 10.1111/j.1365-3180.2010.00820.x

[47] Khaket TP, Singh M, Dhanda S, Singh T, Singh J. Biochemical characterization of consortium compost of toxic weeds *Parthenium hysterophorus* and *Eichhornia crassipe*. Bioresour Technol. 2012;123:360–5. doi: 10.1016/j.biortech.2012.07.107 22940342

[48] Rawat I, Suthar S. Composting of tropical toxic weed *Lantana camera* L. biomass and its suitability for agronomic applications. Compost Science & Utilization. 2014;22(3):105–15. doi: 10.1080/1065657x.2014.895455

[49] Yost JL, Hartemink AE. Soil organic carbon in sandy soils: a review. Advances in Agronomy. 2019 217–310. doi: 10.1016/bs.agron.2019.07.004

[50] Hafez EM, Omara AED, Alhumaydhi FA, El-Esawi MA. Minimizing hazard impacts of soil salinity and water stress on wheat plants by soil application of vermicompost and biochar. Physiol Plant. 2021;172(2):587–602. doi: 10.1111/ppl.13261 33159342

[51] Qu W, Han G, Wang J, Li J, Zhao M, He W, et al. Short-term effects of soil moisture on soil organic carbon decomposition in a coastal wetland of the Yellow River Delta. Hydrobiologia. 2020;848(14):3259–71. doi: 10.1007/s10750-020-04422-8

[52] Araya A, Prasad PVV, Ciampitti IA, Jha PK. Using crop simulation model to evaluate influence of water management practices and multiple cropping systems on crop yields: A case study for Ethiopian highlands. Field Crops Research. 2021;260:108004. doi: 10.1016/j.fcr.2020.108004

[53] Ndung’u M, Mugwe JN, Mucheru-Muna MW, Ngetich FK, Mairura FS, Mugendi DN. Tied-ridging and soil inputs enhance small-scale maize productivity and profitability under erratic rainfall conditions in central Kenya. Agricultural Water Management. 2023;286:108390. doi: 10.1016/j.agwat.2023.108390

[54] Wang D, Zhao P, Xiang R, He S, Zhou Y, Yin X, et al. Nitrogen fertilization overweighs intercropping in promotion of dissolved organic carbon concentration and complexity in potato-cropped soil. Plant Soil. 2021;462(1–2):273–84. doi: 10.1007/s11104-021-04876-2

[55] Peng Y, Xu H, Wang Z, Shi J, Lv J, Wang X. Responses of the content and spectral characteristics of dissolved organic matter in intercropping soil to drought in northeast China. Plant Soil. 2023;506(1–2):471–85. doi: 10.1007/s11104-023-05931-w

[56] Cong W-F, Hoffland E, Li L, Janssen BH, van der Werf W. Intercropping affects the rate of decomposition of soil organic matter and root litter. Plant Soil. 2015;391(1–2):399–411. doi: 10.1007/s11104-015-2433-5

[57] Li Q, Wu L, Chen J, Khan MA, Luo X, Lin W. Biochemical and microbial properties of rhizospheres under maize/peanut intercropping. J Integr Agric. 2016;15(1):101–10. doi: 10.1016/s2095-3119(15)61089-9

[58] Xiao X, Han L, Chen H, Wang J, Zhang Y, Hu A. Intercropping enhances microbial community diversity and ecosystem functioning in maize fields. Front Microbiol. 2023;13:1084452. doi: 10.3389/fmicb.2022.1084452 36687629 PMC9846038

[59] Liu M, Zhao H. Maize-soybean intercropping improved maize growth traits by increasing soil nutrients and reducing plant pathogen abundance. Front Microbiol. 2023;14:1290825. doi: 10.3389/fmicb.2023.1290825 38098655 PMC10720616

[60] Duchene O, Vian J-F, Celette F. Intercropping with legume for agroecological cropping systems: Complementarity and facilitation processes and the importance of soil microorganisms. A review. Agric Ecosyst Environ. 2017;240:148–61. doi: 10.1016/j.agee.2017.02.019

[61] Li Q, Chen J, Wu L, Luo X, Li N, Arafat Y, et al. Belowground Interactions Impact the Soil Bacterial Community, Soil Fertility, and Crop Yield in Maize/Peanut Intercropping Systems. Int J Mol Sci. 2018;19(2):622. doi: 10.3390/ijms19020622 29470429 PMC5855844

[62] Ghanbari A, Dahmardeh M, Siahsar BA, Ramroudi M. Effect of maize (*Zea mays* L.) - cowpea (*Vigna unguiculata* L.) intercropping on light distribution, soil temperature and soil moisture in arid environment. J Food Agric Sci. 2010;8(1):102–8.

[63] Ghosh PK, Bandyopadhyay KK, Wanjari RH, Manna MC, Misra AK, Mohanty M, et al. Legume effect for enhancing productivity and nutrient use-efficiency in major cropping systems–an Indian perspective: a review. Journal of Sustainable Agriculture. 2007;30(1):59–86. doi: 10.1300/j064v30n01_07

[64] Adhikary S. Vermicompost, the story of organic gold: a review. AS. 2012;03(07):905–17. doi: 10.4236/as.2012.37110

[65] Taye T, Ejeta G. Sorghum Production in Transition Through Striga Management. vol. 1. Addis Ababa, Ethiopia: EIAR (Ethiopian Institute of Agricultural Research); 2019.

[66] Mrema E, Shimelis H, Laing M, Mwadzingeni L. Integrated management of *Striga hermonthica* and *S. asiatica* in sorghum: a review. Aust J Crop Sci. 2020;14(01):36–45. doi: 10.21475/ajcs.20.14.01.p1749

[67] Ekeleme F, Jibrin JM, Kamara AY, Oluoch M, Samndi AM, Fagge AA. Assessment of the relationship between soil properties, *Striga hermonthica* infestation and the on-farm yields of maize in the dry Savannas of Nigeria. Crop Protection. 2014;66:90–7. doi: 10.1016/j.cropro.2014.09.001

[68] Yang L, Zhao F, Chang Q, Li T, Li F. Effects of vermicomposts on tomato yield and quality and soil fertility in greenhouse under different soil water regimes. Agricultural Water Management. 2015;160:98–105. doi: 10.1016/j.agwat.2015.07.002

[69] Liu C, Feng X, Xu Y, Kumar A, Yan Z, Zhou J, et al. Legume-based rotation enhances subsequent wheat yield and maintains soil carbon storage. Agron Sustain Dev. 2023;43(5). doi: 10.1007/s13593-023-00918-4

[70] Singh A, Karmegam N, Singh GS, Bhadauria T, Chang SW, Awasthi MK, et al. Earthworms and vermicompost: an eco-friendly approach for repaying nature’s debt. Environ Geochem Health. 2020;42(6):1617–42. doi: 10.1007/s10653-019-00510-4 31974693

[71] Chatterjee R, Debnath A, Mishra S. Vermicompost and Soil Health. In Soil Health, no. 59, Giri B, Varma A, editors. Switzerland AG: Springer Nature Switzerland; 2020. p. 69–88.

[72] Alemayehu S, Kaba F, Taddesse N, Dechassa S, Jj S. Effect of vermicompost and nitrogen application on striga incidence, growth, and yield of sorghum *Sorghum bicolor* (L.) monech in Fedis, eastern Ethiopia. Int J Life Sci. 2016;4(3):349–60.

[73] Bekele G, Dechassa N, Tana T, Sharma JJ. Effects of nitrogen, phosphorus and vermicompost fertilizers on productivity of groundnut (*Arachis hypogaea* L.) in Babile, Eastern Ethiopia. Agron Res. 2019;17(4):1532–46.

[74] Mesfin T, Tesfahunegn GB, Wortmann CS, Nikus O, Mamo M. Tied-ridging and fertilizer use for sorghum production in semi-arid Ethiopia. Nutr Cycl Agroecosyst. 2009;85(1):87–94. doi: 10.1007/s10705-009-9250-2

[75] Nkoa R, Owen MDK, Swanton CJ. Weed abundance, distribution, diversity, and community analyses. Weed sci. 2015;63(SP1):64–90. doi: 10.1614/ws-d-13-00075.1

[76] Travlos IS, Cheimona N, Roussis I, Bilalis DJ. Weed-species abundance and diversity indices in relation to tillage systems and fertilization. Front Environ Sci. 2018;6. doi: 10.3389/fenvs.2018.00011

[77] Przemieniecki SW, Zapałowska A, Skwiercz A, Damszel M, Telesiński A, Sierota Z, et al. An evaluation of selected chemical, biochemical, and biological parameters of soil enriched with vermicompost. Environ Sci Pollut Res Int. 2021;28(7):8117–27. doi: 10.1007/s11356-020-10981-z 33051843 PMC7854409

[78] Wogi L, Dechassa N, Haileselassie B, Mekuria F, Abebe A, Tamene L. A guide to standardized methods of analysis for soil, water, plant, and fertilizer resources for data documentation and knowledge sharing in Ethiopia. 2021.

[79] Chimdessa T. Amelioration of acidic nitisols using lime and vermicompost in Negasa Area, East Wollega Zone, Ethiopia. IJEES. 2021;6(1):1. doi: 10.11648/j.ijees.20210601.11

[80] Chemeda M. Effect of mulching and tied ridge on crop production and soil improvement in dry land areas. JENR. 2022;6(2). doi: 10.23880/jenr-16000275

[81] Abie Y, Reda Y, Lamesign H, Esubalew T. Effect of ridging and tie-ridging time on yield and yield component of sorghum in Northern Ethiopia. Heliyon. 2024;10(5):e26817. doi: 10.1016/j.heliyon.2024.e26817 38449638 PMC10915371

[82] Naikwade PV. Soil organic carbon sequestration by long-term application of manures prepared from *Trianthema portulacastrurm* Linn. Communications in Soil Science and Plant Analysis. 2019;50(20):2579–92. doi: 10.1080/00103624.2019.1671442

[83] Layek J. “Cereal+Legume Intercropping: An Option for Improving Productivity and Sustaining Soil Health,” in Legumes for Soil Health and Sustainable Management, Meena R. S., editor. Springer Nature Singapore Pte Ltd.; 2018. p. 347–86.

[84] Demir Z. Effects of Vermicompost on Soil Physicochemical Properties and Lettuce (*Lactuca sativa* Var. Crispa) Yield in Greenhouse under Different Soil Water Regimes. Communications in Soil Science and Plant Analysis. 2019;50(17):2151–68. doi: 10.1080/00103624.2019.1654508

[85] Angelova V, Akova VI, Artinova NS, Ivanov KI. The effect of organic amendments on soil chemical characteristics. Bulg J Agric Sci. 2013;19(5):958–71.

[86] Atsbha G, Taye T, Ibrahim H, Demeke N. Striga infestation and its interaction to density of sorghum, basic chemical and physical properties of the soil across Tigray Region, Northern Ethiopia. ISABB J Food and Agric Sci. 2018;8(1):1–9. doi: 10.5897/isabb-jfas2016.0058

[87] Li X. Effects of different organic fertilizers on improving soil from newly reclaimed land to crop soil. Agric. 2021;11(560):1–15.

[88] Phiri AT, Njira KOW. Grain legume-based cropping systems’ effects on soil organic carbon and nutrient dynamics. Agric Res. 2022. doi: 10.1007/s40003-022-00619-6

[89] Nasar J, Ahmad M, Gitari H, Tang L, Chen Y, Zhou X-B. Maize/soybean intercropping increases nutrient uptake, crop yield and modifies soil physio-chemical characteristics and enzymatic activities in the subtropical humid region based in Southwest China. BMC Plant Biol. 2024;24(1):434. doi: 10.1186/s12870-024-05061-0 38773357 PMC11106902

[90] Attallah A, Hamdi W, Souid A, Farissi M, Boulbaba L, Messiga AJ. Impact of cereal-legume intercropping on changes in soil nutrients contents under semi-arid conditions. Sustain. 2024;16(2725):1–12.

[91] Arancon NQ, Edwards CA, Bierman P. Influences of vermicomposts on field strawberries: part 2. Effects on soil microbiological and chemical properties. Bioresour Technol. 2006;97(6):831–40. doi: 10.1016/j.biortech.2005.04.016 15979873

[92] Adebayo A, Wahab A, Lawal OO. Impact of vermicompost and its residual effect on soil properties, nutrient uptake and yield of cucumber (*Cucumis sativus* L.). Inter J Organ Argic Res Dev. 2021;17:1–19.

[93] Wang F, Wang X, Song N. Biochar and vermicompost improve the soil properties and the yield and quality of cucumber (*Cucumis sativus* L.) grown in plastic shed soil continuously cropped for different years. Agric Ecosyst Environ. 2021;315:107425. doi: 10.1016/j.agee.2021.107425

[94] Layek J, Das A, Mitran T, Remote N, Centre S, Meena RS. Cereal+Legume Intercropping: An Option for Improving Productivity and Sustaining Soil Health. In: Legumes for Soil Health and Sustainable Management. Das RSMA, Lal GSYR, editors. Singapore: Springer Nature; 2018. p. 347–86.

[95] Guerchi A, Mnafgui W, Jabri C, Merghni M, Sifaoui K, Mahjoub A. Improving productivity and soil fertility in *Medicago sativa* and *Hordeum marinum* through intercropping under saline conditions. BMC Plant Biology. 2024;24(158):1–14. 38429693 10.1186/s12870-024-04820-3 PMC10905945

[96] Lim SL, Wu TY, Lim PN, Shak KPY. The use of vermicompost in organic farming: overview, effects on soil and economics. J Sci Food Agric. 2015;95(6):1143–56. doi: 10.1002/jsfa.6849 25130895

[97] Zhang F, Wang R, Yu W, Liang J, Liao X. Influences of a vermicompost application on the phosphorus transformation and microbial activity in a paddy soil. Soil Water Res. 2020;15(4):199–210. doi: 10.17221/91/2019-swr

[98] Negese W, Wogi L, Geleto T. Responses of acidic soil to lime and Vermicompost Application at Lalo Asabi District, Western Ethiopia. SR. 2021;9(6):108. doi: 10.11648/j.sr.20210906.12

[99] Zarate-Valdez JL, Zasoski RJ, Lauchli AE. Short-term effects of moisture content on soil solution pH and soil redox potential (Eh). Soil Science. 2006;171(5):423–31. doi: 10.1097/01.ss.0000222887.13383.08

[100] Saha MK, Khan MHR, Akter S, Hossain MB. Integrated effects of vermicompost, climatic factors and soil mixing on selected soil fertility indicators. Dhaka Univ J Biol Sci. 2023;32(1):119–34. doi: 10.3329/dujbs.v32i1.64196

[101] Yagi R, Ferreira ME, Cruz MCP da, Barbosa JC. Organic matter fractions and soil fertility under the influence of liming, vermicompost and cattle manure. Sci agric (Piracicaba, Braz). 2003;60(3):549–57. doi: 10.1590/s0103-90162003000300021

[102] Kaiser M, Ellerbrock RH, Gerke HH. Cation exchange capacity and composition of soluble soil organic matter fractions. Soil Science Soc of Amer J. 2008;72(5):1278–85. doi: 10.2136/sssaj2007.0340

[103] Goswami L, Nath A, Sutradhar S, Bhattacharya SS, Kalamdhad A, Vellingiri K, et al. Application of drum compost and vermicompost to improve soil health, growth, and yield parameters for tomato and cabbage plants. J Environ Manage. 2017;200:243–52. doi: 10.1016/j.jenvman.2017.05.073 28582747

[104] Esteves G de F, de Souza KRD, Bressanin LA, Andrade PCC, Veroneze Júnior V, Dos Reis PE, et al. Vermicompost improves maize, millet and sorghum growth in iron mine tailings. J Environ Manage. 2020;264:110468. doi: 10.1016/j.jenvman.2020.110468 32250898

[105] Demir Z. Alleviation of Adverse Effects of Sodium on Soil Physicochemical Properties by Application of Vermicompost. Compost Science & Utilization. 2020;28(2):100–16. doi: 10.1080/1065657x.2020.1789011

[106] Mengistu T, Gebrekidan H, Kibret K, Woldetsadik K, Shimelis B, Yadav H. The integrated use of excreta-based vermicompost and inorganic NP fertilizer on tomato (Solanum lycopersicum L.) fruit yield, quality and soil fertility. Int J Recycl Org Waste Agricult. 2017;6(1):63–77. doi: 10.1007/s40093-017-0153-y

[107] Ncube B, Dimes JP, van Wijk MT, Twomlow SJ, Giller KE. Productivity and residual benefits of grain legumes to sorghum under semi-arid conditions in south-western Zimbabwe: Unravelling the effects of water and nitrogen using a simulation model. Field Crops Research. 2009;110(2):173–84. doi: 10.1016/j.fcr.2008.08.001

[108] Senaratne R, Liyanage NDL, Soper RJ. Nitrogen fixation of and N transfer from cowpea, mungbean and groundnut when intercropped with maize. Fertil Res. 1995;40:41–8.

[109] Wang Z-G, Jin X, Bao X-G, Li X-F, Zhao J-H, Sun J-H, et al. Intercropping enhances productivity and maintains the most soil fertility properties relative to sole cropping. PLoS One. 2014;9(12):e113984. doi: 10.1371/journal.pone.0113984 25486249 PMC4259307

[110] Tang X. Cassava/peanut intercropping improves soil quality via rhizospheric microbes increased available nitrogen contents. BMC Biotechn. 2020;20:1–11. 10.1186/s12896-020-00606-1 PMC7049180 32111197

[111] Bader BR, Taban SK, Fahmi AH, Abood MA, Hamdi GJ. Potassium availability in soil amended with organic matter and phosphorous fertiliser under water stress during maize (*Zea mays* L) growth. Journal of the Saudi Society of Agricultural Sciences. 2021;20(6):390–4. doi: 10.1016/j.jssas.2021.04.006

[112] Yen Y-S, Chen K-S, Yang H-Y, Lai H-Y. Effect of Vermicompost Amendment on the Accumulation and Chemical Forms of Trace Metals in Leafy Vegetables Grown in Contaminated Soils. Int J Environ Res Public Health. 2021;18(12):6619. doi: 10.3390/ijerph18126619 34205439 PMC8296319

[113] Xue J, Bakker MR, Milin S, Graham D. Enhancement in soil fertility, early plant growth and nutrition and mycorrhizal colonization by vermicompost application varies with native and exotic tree species. J Soils Sediments. 2022;22(6):1662–76. doi: 10.1007/s11368-022-03180-5

[114] Akhter S, Sen R, Akter S, Teixeira JA, Haque A, Noor S. Efficacy of vermicompost to improve soil health, yield and nutrient uptake of cauliflower in grey terrace soil of Bangladesh. Dyn Soil, Dyn Plant. 2012;6(1):103–9.

[115] Bekele A, Kibret K, Bedadi B, Yli-halla M, Balemi T. Effects of lime, vermicompost, and chemical P fertilizer on selected properties of acid soils of Ebantu District, Western Highlands of Ethiopia. Applied Environmental Soil Science. 2018;2018:1–13.

[116] White PJ, Brown PH. Plant nutrition for sustainable development and global health. Ann Bot. 2010;105(7):1073–80. doi: 10.1093/aob/mcq085 20430785 PMC2887071

[117] Asrin AFN, Hanom SAK, Ossain SHAKH. Effects of vermicompost and compost on soil properties and growth and yield of Kalmi (Ipomoea aquatica Forsk.) in mixed soil. Dhaka Univ J Biol Sci. 2019;28(1):121–9.

[118] Gacheru E, Rao MR. Managing strigainfestation on maize using organic and inorganic nutrient sources in western Kenya. International Journal of Pest Management. 2001;47(3):233–9. doi: 10.1080/09670870110044616

[119] Goldwasser Y, Rodenburg J. Integrated Agronomic Management of Parasitic Weed Seed Banks. In Parasitic Orobanchaceae Parasitic Mechanisms and Control Strategies. Berlin Heidelberg: Springer-Verlag; 2013. p. 393–413.

[120] Ayongwa GC, Stomph TJ, Belder P, Leffelaar PA, Kuyper TW. Organic matter and seed survival of *Striga hermonthica* – Mechanisms for seed depletion in the soil. Crop Protection. 2011;30(12):1594–600. doi: 10.1016/j.cropro.2011.08.012

[121] Jamil M, Kanampiu FK, Karaya H, Charnikhova T, Bouwmeester HJ. *Striga hermonthica* parasitism in maize in response to N and P fertilisers. Field Crops Research. 2012;134:1–10. doi: 10.1016/j.fcr.2012.03.015

[122] Gebremedhin Woldemariam Z, Alemayehu Damot G, Ayalew Zewdie D. Nitrogen fertilizer and cattle manure for Striga (*Striga hermonthica*) management and enhancement of sorghum productivity in northwest Ethiopia. Journal of Plant Nutrition. 2021;45(2):232–45. doi: 10.1080/01904167.2021.1952219

[123] Abunyewa AA, Padi FK. Changes in soil fertility and *Striga hermonthica* prevalence associated with legume and cereal cultivation in the Sudan savannah zone of Ghana. Land Degrad Dev. 2003;14(3):335–43. doi: 10.1002/ldr.555

[124] Dhaliwal SS, Sharma V, Shukla AK, Gupta RK, Verma V. Residual effect of organic and inorganic fertilizers on growth, yield and nutrient uptake in wheat under a basmati rice – wheat cropping system in north-western India. Agric. 2023;13(556):1–17.

[125] Mak-Mensah E, Obour PB, Wang Q. Influence of tied-ridge-furrow with inorganic fertilizer on grain yield across semiarid regions of Asia and Africa: A meta-analysis. PeerJ. 2021;9:e11904. doi: 10.7717/peerj.11904 34458020 PMC8378338

[126] Varatharajan T, Dass A, Choudhary AK, Pooniya V, Das TK, Dhar S, et al. Residual effect of maize (*Zea mays*) + blackgram (Vigna mungo) intercropping on growth, factor productivity and resource-use efficiency of succeeding wheat (Triticum aestivum) under integrated crop management. IJA. 2023;67(4):392–400. doi: 10.59797/ija.v67i4.144

[127] Zhao X, Dong Q, Han Y, Zhang K, Shi X, Yang X, et al. Maize/peanut intercropping improves nutrient uptake of side-row maize and system microbial community diversity. BMC Microbiol. 2022;22(1):14. doi: 10.1186/s12866-021-02425-6 34996375 PMC8740425

