# Supplementary material for: Residual effect of vermicompost and preceding groundnut on soil fertility and associated Striga density under sorghum cropping in Eastern Ethiopia
Source: PLoS One. 2025 Mar 12;20(3):e0318057. doi: 10.1371/journal.pone.0318057 (PMC11903043; doi:10.1371/journal.pone.0318057)
Supplement: S1 File — S1 Table. Soil pH as influenced by Vermicompost with cropping systems [seedbed types (A) and sowing methods (B)]. S2 Table. Soil organic carbon (SOC) as influenced by Vermicompost with sowing methods. S3 Table. Potassium (K) as influenced by Vermicompost with sowing methods. S4 Table. Soil Calcium (Ca) as influenced by Vermicompost with sowing methods. S5 Table. Soil Magnesium (Mg) as influenced by Vermicompost with sowing methods. S6 Table. Striga density as influenced by Vermicompost with cropping systems. (DOCX) [file pone.0318057.s001.docx]

# Supplemental information files were used illustrate data form which each figure derived except Fig 1.

# Table S1: Soil pH as influenced by Vermicompost with cropping systems [seedbed types (A) and sowing methods (B)]

A.

| Factors | | N | pH | |
| --- | --- | --- | --- | --- |
| SB | **VC** |  | **Mean** | **Std Dev** |
| F | **V0** | **6** | 7.02500000 | 0.09731393 |
| F | **V1** | **6** | 7.11666667 | 0.05750362 |
| F | **V2** | **6** | 7.32166667 | 0.02786874 |
| F | **V3** | **6** | 7.29166667 | 0.11373947 |
| TD | **V0** | **6** | 6.89833333 | 0.12155931 |
| TD | **V1** | **6** | 7.19000000 | 0.05059644 |
| TD | **V2** | **6** | 7.27166667 | 0.03816630 |
| TD | **V3** | **6** | 7.21333333 | 0.08140434 |

NB: Vermicompost (VC) with seedbed (SB) types (F=furrowing; TD=tied-ridge); N= Class Level; Std Dev=standard deviation

B.

| Factors | | N | pH | |
| --- | --- | --- | --- | --- |
| SM | **VC** |  | **Mean** | **Std Dev** |
| SB2 | **V0** | **6** | 6.92333333 | 0.07229569 |
| SB2 | **V1** | **6** | 7.15833333 | 0.05455884 |
| SB2 | **V2** | **6** | 7.28000000 | 0.03405877 |
| SB2 | **V3** | **6** | 7.17166667 | 0.04875107 |
| SS | **V0** | **6** | 7.00000000 | 0.15849290 |
| SS | **V1** | **6** | 7.14833333 | 0.07782459 |
| SS | **V2** | **6** | 7.31333333 | 0.04366539 |
| SS | **V3** | **6** | 7.33333333 | 0.07201852 |

**NB:** Vermicompost (VC) with sowing methods (SM) (SS= sole sorghum SB2=intercropped sorghum); N= Class Level; Std Dev=standard deviation

# Table S2: Soil organic carbon (SOC) as influenced by Vermicompost with sowing methods

| Factors | | N | SOC | |
| --- | --- | --- | --- | --- |
| SM | **VC** |  | **Mean** | **Std Dev** |
| SB2 | **V0** | **6** | 0.80000000 | 0.05215362 |
| SB2 | **V1** | **6** | 1.14833333 | 0.12188793 |
| SB2 | **V2** | **6** | 1.52833333 | 0.06369197 |
| SB2 | **V3** | **6** | 2.42500000 | 0.10193135 |
| SS | **V0** | **6** | 0.74500000 | 0.04370355 |
| SS | **V1** | **6** | 1.12500000 | 0.05089204 |
| SS | **V2** | **6** | 1.52166667 | 0.08635199 |
| SS | **V3** | **6** | 2.19000000 | 0.04049691 |

**NB:** Vermicompost (VC) with sowing methods (SM) (SS= sole sorghum SB2=intercropped sorghum); N= Class Level; Std Dev=standard deviation

# Table S3: Potassium (K) as influenced by Vermicompost with sowing methods

| \| Factors \| \| N \| K \| \| \| --- \| --- \| --- \| --- \| --- \| \| SM \| **VC** \| **Mean** \| **Std Dev** \| \| SB2 \| **V0** \| **6** \| 0.35666667 \| 0.04320494 \| \| SB2 \| **V1** \| **6** \| 0.45666667 \| 0.06562520 \| \| SB2 \| **V2** \| **6** \| 0.74833333 \| 0.06047038 \| \| SB2 \| **V3** \| **6** \| 1.09833333 \| 0.04070217 \| \| SS \| **V0** \| **6** \| 0.24166667 \| 0.06080022 \| \| SS \| **V1** \| **6** \| 0.41500000 \| 0.04969909 \| \| SS \| **V2** \| **6** \| 0.67333333 \| 0.03614784 \| \| SS \| **V3** \| **6** \| 0.90000000 \| 0.06723095 \| |  |  |  | |
| --- | --- | --- | --- | --- | --- | --- | --- | --- | --- | --- | --- | --- | --- | --- | --- | --- | --- | --- | --- | --- | --- | --- | --- | --- | --- | --- | --- | --- | --- | --- | --- | --- | --- | --- | --- | --- | --- | --- | --- | --- | --- | --- | --- | --- | --- | --- | --- | --- | --- | --- | --- | --- | --- |
|  |  |  |  |  |
|  |  |  |  |  |

**NB:** Vermicompost (VC) with sowing methods (SM) (SS= sole sorghum SB2=intercropped sorghum); N= Class Level; Std Dev=standard deviation

# Table S4: Soil Calcium (Ca) as influenced by Vermicompost with sowing methods

| Factors | | N | Ca | |
| --- | --- | --- | --- | --- |
| SM | **VC** |  | **Mean** | **Std Dev** |
| SB2 | **V0** | **6** | 9.7500000 | 1.16629327 |
| SB2 | **V1** | **6** | 12.3916667 | 1.74654421 |
| SB2 | **V2** | **6** | 15.3633333 | 1.36694794 |
| SB2 | **V3** | **6** | 19.6650000 | 1.22632377 |
| SS | **V0** | **6** | 8.9800000 | 0.87452844 |
| SS | **V1** | **6** | 13.1433333 | 2.19841458 |
| SS | **V2** | **6** | 15.8533333 | 1.60154509 |
| SS | **V3** | **6** | 17.9216667 | 1.03875727 |

**NB:** Vermicompost (VC) with sowing methods (SM) (SS= sole sorghum SB2=intercropped sorghum); N= Class Level; Std Dev=standard deviation

# Table S5: Soil Magnesium (Mg) as influenced by Vermicompost with sowing methods

| Factors | | N | Mg | |
| --- | --- | --- | --- | --- |
| SM | **VC** |  | **Mean** | **Std Dev** |
| SB2 | **V0** | **6** | 3.17500000 | 0.21961330 |
| SB2 | **V1** | **6** | 3.35166667 | 0.18573278 |
| SB2 | **V2** | **6** | 4.24666667 | 0.14610499 |
| SB2 | **V3** | **6** | 4.94500000 | 0.21454603 |
| SS | **V0** | **6** | 2.84833333 | 0.16916461 |
| SS | **V1** | **6** | 3.35833333 | 0.12671490 |
| SS | **V2** | **6** | 4.05333333 | 0.12110601 |
| SS | **V3** | **6** | 4.44833333 | 0.21348692 |

**NB:** Vermicompost (VC) with sowing methods (SM) (SS= sole sorghum SB2=intercropped sorghum); N= Class Level; Std Dev=standard deviation

# Table S6: *Striga* density as influenced by Vermicompost with cropping systems

| Factors | | | N | Density | |
| --- | --- | --- | --- | --- | --- |
| SM | **SB** | **VC** |  | **Mean** | **Std Dev** |
| SB2 | **F** | **V0** | **3** | 8.1033333 | 0.56500737 |
| SB2 | **F** | **V1** | **3** | 5.1433333 | 0.65592174 |
| SB2 | **F** | **V2** | **3** | 3.7666667 | 0.68966175 |
| SB2 | **F** | **V3** | **3** | 1.9533333 | 0.34645827 |
| SB2 | **TD** | **V0** | **3** | 6.1033333 | 0.08326664 |
| SB2 | **TD** | **V1** | **3** | 3.2400000 | 0.08717798 |
| SB2 | **TD** | **V2** | **3** | 2.0000000 | 0.07810250 |
| SB2 | **TD** | **V3** | **3** | 1.0933333 | 0.08386497 |
| SS | **F** | **V0** | **3** | 12.9033333 | 0.14294521 |
| SS | **F** | **V1** | **3** | 8.4966667 | 0.43558390 |
| SS | **F** | **V2** | **3** | 5.4866667 | 0.24440404 |
| SS | **F** | **V3** | **3** | 3.5700000 | 0.32924155 |
| SS | **TD** | **V0** | **3** | 7.8500000 | 0.12124356 |
| SS | **TD** | **V1** | **3** | 5.4666667 | 0.43061971 |
| SS | **TD** | **V2** | **3** | 3.9266667 | 0.26576932 |
| SS | **TD** | **V3** | **3** | 1.6733333 | 0.26689573 |

**NB:** Vermicompost (VC) with sowing methods (SM) (SS= sole sorghum SB2=intercropped sorghum) and seedbed (SB) types (F=furrowing; TD=tied-ridge); N= Class Level; Std Dev=standard deviation
